# Insights into the Mechanism of Bovine Spermiogenesis Based on Comparative Transcriptomic Studies

**DOI:** 10.3390/ani11010080

**Published:** 2021-01-05

**Authors:** Xin Li, Chenying Duan, Ruyi Li, Dong Wang

**Affiliations:** 1 Institute of Animal Science, Chinese Academy of Agricultural Sciences, Beijing 100193, China; lixinxinli_sadu@163.com; 2 College of Animal Science and Technology, Jilin Agricultural University, Changchun 130118, China; duan19970103@163.com (C.D.); lry19961209@163.com (R.L.)

**Keywords:** spermiogenesis, differentially expressed genes, homology trends analysis

## Abstract

**Simple Summary:**

Any irregularity in spermiogenesis reduces the quality of semen and may lead to male sterility in cattle and humans. Thus, we investigated the differential transcriptomics of spermatids from round spermatid to epididymal sperm and compared them with the transcriptomics of mice in the same period. We found differentially expressed genes (DEGs) involved in sperm head and tail formation, and epigenetic regulatory networks which regulated genetic material condensation, the deformation of the spermatid, and the expression of genes in it. According to the sterility report on the ART3 protein and its possible epigenetic function, we detected that it was localised outside the spermatocyte, in round and elongated spermatids. Interestingly, we observed that the ART3 protein on round and elongated spermatids was localised approximately to the lumen of seminiferous tubule. It was also localised on the head and tail part near the head in epididymal sperm, suggesting its important role in the deformation from round spermatids to sperm. Our findings provide new insights into the molecular mechanism underlying bovine spermiogenesis, thereby contributing to the improved reproductive potential of cattle and the development of strategies for the diagnosis and treatment of male infertility.

**Abstract:**

To reduce subfertility caused by low semen quality and provide theoretical guidance for the eradication of human male infertility, we sequenced the bovine transcriptomes of round, elongated spermatids and epididymal sperms. The differential analysis was carried out with the reference of the mouse transcriptome, and the homology trends of gene expression to the mouse were also analysed. First, to explore the physiological mechanism of spermiogenesis that profoundly affects semen quality, homological trends of differential genes were compared during spermiogenesis in dairy cattle and mice. Next, Gene Ontology (GO), Kyoto Encyclopaedia of Genes and Genomes (KEGG) pathway enrichment, protein–protein interaction network (PPI network), and bioinformatics analyses were performed to uncover the regulation network of acrosome formation during the transition from round to elongated spermatids. In addition, processes that regulate gene expression during spermiogenesis from elongated spermatid to epididymal sperm, such as ubiquitination, acetylation, deacetylation, and glycosylation, and the functional *ART3* gene may play important roles during spermiogenesis. Therefore, its localisation in the seminiferous tubules and epididymal sperm were investigated using immunofluorescent analysis, and its structure and function were also predicted. Our findings provide a deeper understanding of the process of spermiogenesis, which involves acrosome formation, histone replacement, and the fine regulation of gene expression.

## 1. Introduction

With advancements in artificial insemination, breeders progressively focused on semen requirements for the quality and quantity of excellent bovines. Approximately 10 to 60 billion sperms can be obtained from bovines when semen is collected three times a week [1]. Considering that there are 20 million sperms per straw and a calving rate of 53% [2], each bovine can theoretically breed approximately 13,000 to 78,000 offspring yearly. However, owing to semen quality issues caused by insufficient spermiogenesis, the estimated pregnancy rate of each straw of frozen semen varies by 20% [2], resulting in a tremendous waste. In addition, approximately 15% of couples of childbearing age worldwide are affected by infertility, of which male factors account for 50% [3]. Sperm with normal morphology can also cause infertility [4]. The quality standard for frozen bovine semen (GB4143-2008) stipulates that the proportion of morphologically abnormal spermatozoa in qualified bovine semen should be less than 18%, while the Real-World Health Organization Human Semen Analysis Laboratory Technical Manual (Fifth Edition) stipulates that that semen should be less than 4% in humans. Therefore, it is necessary to generate new insights on the mechanisms underlying spermiogenesis, identify important regulatory pathways and functional genes to improve the semen quality and yield of herd sire to eventually overcome male infertility [2,5].

**Figure 1:**
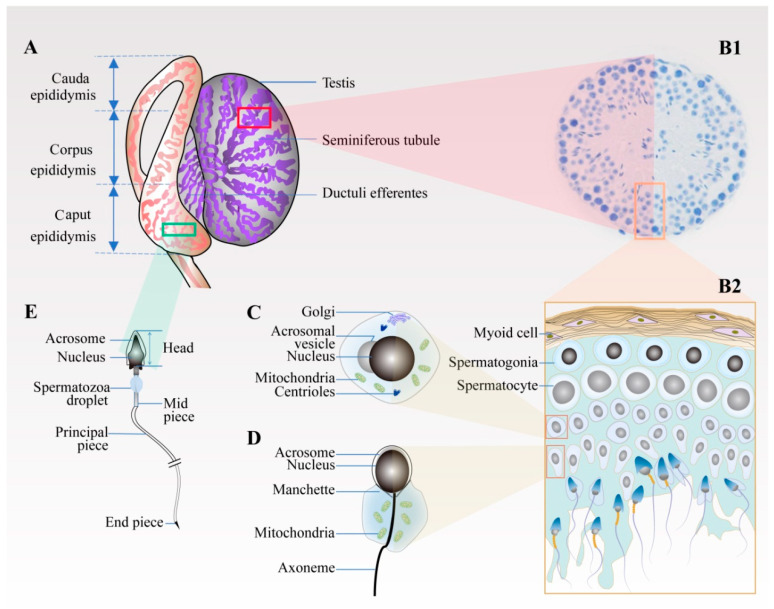
Schematic diagram of spermiogenesis in the testis and epididymis. (**A**) Testis and epididymis. Spermatogenesis mainly occurs on the spermatogenic epithelium inside the long and coiled seminiferous tubules comprising the testis. After leaving the testicle, the deformed sperm is stored in the epididymis through the ductuli efferentes testis. The epididymal microenvironment promotes the maturation of the sperm. (**B1**,**B2**) Cross-section of seminiferous tubule. (**B1**,**B2**) The cross-section and enlarged view of the seminiferous tubule, respectively. From the tube wall to the lumen, the spermatogenic cells are arranged in layers according to their types. Spermatogonia, primary spermatocytes, and secondary spermatocytes are located near the wall of the tube, whereas the round spermatids, elongated spermatids, and mature spermatozoa are located near the lumen. (**C**) Round spermatid. The haploid round sperm cells are formed after the second meiosis. (**D**) Elongated spermatid. Round spermatids develop into elongated spermatids through a series of processes, including the gradual aggregation and specialisation of Golgi bodies into acrosomes and the extension of centrioles into tail flagella. (**E**) Epididymal sperm. Spermatozoa acquire their fertilising ability and forward motility properties in the epididymis.

Since histones are gradually replaced by protamine in the process of spermiogenesis, chromosomes are highly condensed, resulting in the overall shrinkage of the nucleus. At the same time, the spermatid gradually polarises, and the Golgi bodies progressively aggregate and transfer to one end of the spermatid, and finally become specialised into the acrosome, forming the sperm head together with the nucleus. The centrioles move in the direction opposite to the head and extend outward to become the skeleton of the sperm tail, while mitochondria gradually specialise to form a ring-shaped mitochondrial sheath, which is attached to the outside of the axon filament in the midpiece of the sperm tail. At the same time, the cytoplasmic residue gradually falls off. Following these changes, the round spermatid eventually develops into the tadpole-like sperm with a head and tail, which are mature and stored for a short time in the epididymis (Figure 1).

However, the abnormal expression of important functional genes during spermiogenesis affects the morphology [6] and structure of the sperm and may lead to male sterility [7]. During spermatogenesis, Brdt binds to acetylated histones through the bromo domain to promote the reorganisation of chromatin remodelling and mRNA processing [8]. Its abnormal expression results in dissociation of the acrosome from spermatids in the absence of a tail [9]. Rnf8 mediates the ubiquitination of H2A/H2B, which in turn promotes the substitution of histones with protamine, wherein the deletion of Rnf8 suppresses chromosome condensation, reduces the number of elongated spermatids, and induces abnormal morphology of the sperm head and tail [10]. CatSper glycoproteins form sperm-specific, voltage-gated Ca^2+^ channels localised along the membrane of the sperm flagella. Triggering this channel can increase the probability of sperm–egg binding [11,12].

Additionally, epigenetic modification plays an important role in expression of functional genes during spermiogenesis. In-depth research on epigenetic modifications may help reveal the regulatory mechanism of spermiogenesis and mining important functional genes, and this has become the focus of exploration, prevention, and control of the causes of male breeding difficulty.

Therefore, we investigated the transcriptomic profiles of round spermatids, elongated spermatids, and epididymal sperms of cattle. In order to identify differentially expressed genes that play an important role in spermiogenesis, same-stage mouse transcriptome sequencing data were introduced as a reference. Homology comparison analysis between species was used to obtain bovine genes with the same expression tendency in mice. Through the protein–protein interaction (PPI) network, Gene Ontology (GO) and Kyoto Encyclopaedia of Genes and Genomes (KEGG) pathway enrichment, and bioinformatic analysis of these genes, we initially obtained the protein interaction networks and pathways related to spermiogenesis, such as acrosomal and protamine replacement histones. At the same time, the structure and function of the important functional gene *ART3* were predicted, and the localisation of the ART3 protein in the seminiferous tubules was analysed by immunofluorescence. Our results provide a theoretical basis for future studies aiming to explore the mechanism underlying spermiogenesis and contribute to the improvement of the reproductive potential of cattle and development of human infertility mechanisms.

## 2. Results

### 2.1. Sequencing Analysis of Bovine Spermatids and Sperm

After the quality control of the original sequencing data, the number of clean reads for each sample was more than 66.61 million, which accounted for more than 76.7% of the original sequencing data, and the Clean Q30 Bases ratio of each sample was higher than 91%, indicating that the sequencing data can be used in subsequent experiments (Table 1). After quality control, compared with the bovine reference genome, the alignment ratio of clean reads was at least 86% for each sample (Table 1), suggesting that the sequencing results could cover most of the reference genome. Further analyses could reveal the biological information of the genome during spermiogenesis.

### 2.2. Principal Component Analysis (PCA) Analysis of Bovine and Mouse Transcripts of Spermatids and Sperm

**Figure 2:**
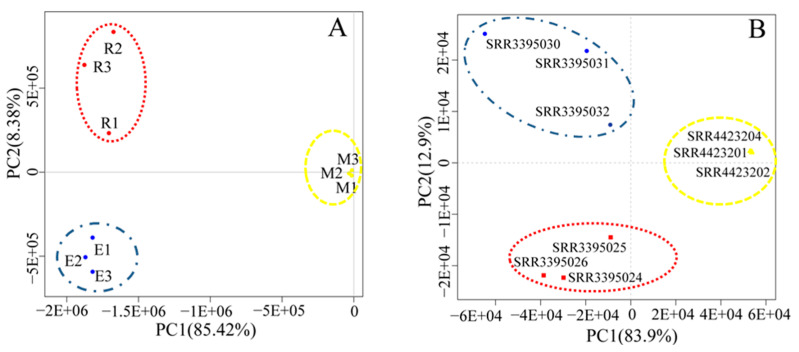
Principal component analysis (PCA) results. Bovine (**A**) and mouse (**B**) PCA graphs of transcript expression in round spermatids, elongated spermatids, and epididymal sperm. PCA plots of RNA-Seq data show the characteristics of samples according to gene expression (FPKM) levels, with each dot indicating a sample. The red dotted circle represents round spermatids, the blue dotted circle represents elongated spermatids, and the yellow dotted circle represents epididymal sperms. The horizontal axis represents the first principal component (PC1), whereas the horizontal axis represents the first principal component (PC2). FPKM: fragments per kilobase of transcript per million mapped reads.

The PCA results in Figure 2A show that the sum of PC1 (first principal component) (67.4%) and PC2 (second principal component) (19.8%) of bovine reached 87.2%, of which round spermatid samples R1, R2, and R3, elongated spermatid samples E1, E2, and E3, and epididymal sperm samples M1, M2, and M3 were clustered together. The PCA results (Figure 2B) showed that the sum of PC1 (83.9%) and PC2 (12.9%) of mice was 96.8%, among which round spermatid samples SRR3395024, SRR3395025, and SRR3395026, elongated spermatid samples SRR3395030, SRR3395031, SRR3395032, and epididymal sperm samples SRR4423201, SRR4423202, and SRR4423204 were clustered together. These results indicate that transcripts of various types of spermatids from bovines and mice showed good intragroup repeatability. In addition, as evidenced by the large differences between the groups, the samples were representative.

### 2.3. Quantitative Real-Time RT PCR (RT-qPCR) Validation of the Bovine RNA-Seq Results

**Figure 3:**
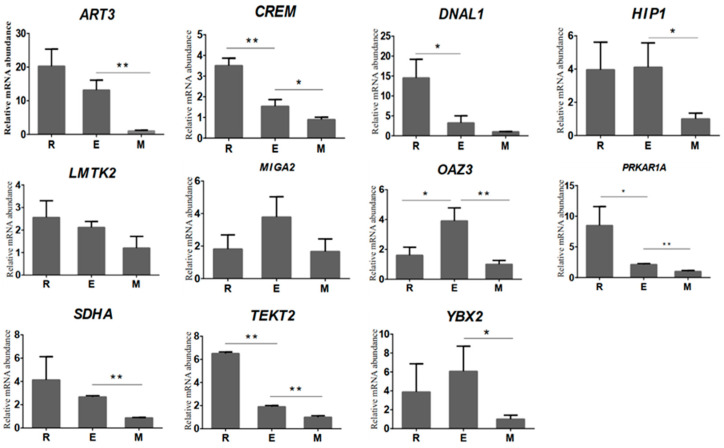
RT-qPCR analysis results of the relative mRNA abundance of 11 genes in bovine round spermatids, elongated spermatids, and epididymal sperm. The horizontal axis represents the type of sample, and the vertical axis represents the relative mRNA abundance normalised to that of β-actin. The relative mRNA abundance of 11 genes was represented as the mean, with error bars indicating standard error of the mean (mean ± SEM). Student’s *t*-test was used to assess the statistical significance of experimental data (* *p* < 0.05, ** *p* < 0.01).

The relative expression of 11 randomly selected genes was higher in round and elongated spermatids than in sperm, which was consistent with the variations obtained from RNA-seq analysis (Figure 3). Among these genes, *LMTK2* and *MIGA2* showed no significant difference (*p* > 0.05) in the sperm deformation stage, *DNAL1* was significantly reduced from round spermatid to elongated spermatid (*p* < 0.05) by 77.7%, and *ART3*, *HIP1*, *SDHA*, and *YBX2* showed significant reduction from elongated spermatid to epididymal spermatid (*p* < 0.05) by 92.4%, 75.7%, 67.6%, and 83.5%. The relative expression levels of *TEKT2* and *PRKAR1A* were significantly reduced (*p* < 0.05) from round spermatid to elongated spermatid by 70.7% and 47.5%, respectively, and elongated spermatid to epididymal sperm (*p* < 0.05 by 75.1% and 52.7%, respectively, *OAZ3* was significantly increased from round to elongated spermatid (*p* < 0.05) by 143.2%, and a significant decline from elongated spermatid to epididymal sperm (*p* < 0.01) by 74.4% was also observed. Our qPCR results validated our RNA-Seq results, indicating that the results of bovine transcriptomic sequencing are reliable and subsequent experimental studies could be carried out. 

### 2.4. Differentially Expressed Genes (DEGs) Screening of the Bovine Spermatids and Sperm

**Figure 4:**
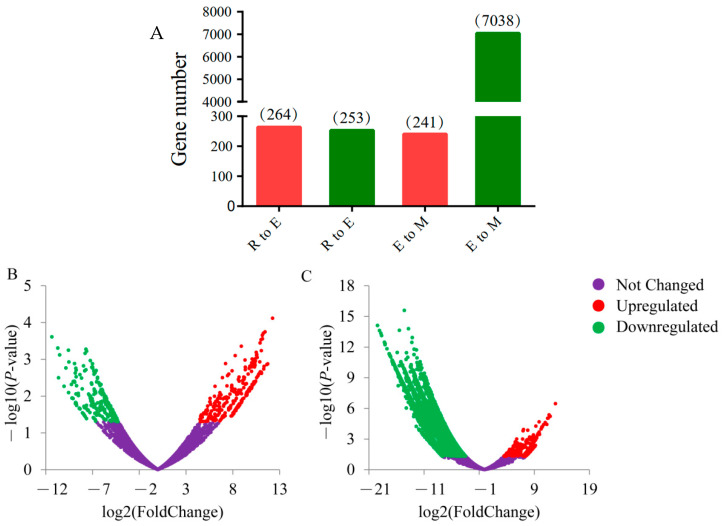
Differentially expressed genes (DEGs) at each deformation stage in bovine sperm. (**A**) Histogram of upregulated and downregulated genes during spermiogenesis. The abscissa R to E represents the stage of round to elongated spermatid, E to M represents the stage of elongated spermatid to epididymal sperm, and the ordinate represents the number of DEGs. (**B**,**C**) DEG volcano diagrams from round spermatid to elongated spermatid and from elongated spermatid to epididymal sperm, respectively. The abscissa represents the differential multiple of genes, whereas the ordinate represents the significance of the difference. *p*-value < 0.05 and |log2 (fold change)| ≥ 1 are used as the screening criteria for DEGs. Red indicates upregulated DEGs, green indicates downregulated DEGs, and purple indicates no significant changes in relative gene expression.

A total of 7652 DEGs were obtained from the differential analysis of the transcriptional expression of genes during spermiogenesis in bovine. Among these, 264 genes were upregulated, and 253 genes were downregulated from round spermatids to elongated spermatids. The number of upregulated genes was slightly higher than that of downregulated genes. From elongated spermatids to epididymal sperm, we identified 241 upregulated and 7038 downregulated genes. The number of upregulated genes was far less than the number of downregulated genes. During spermiogenesis, the number of upregulated genes gradually decreased, whereas that of downregulated genes gradually increased. In particular, at the later stage from elongated spermatids to epididymal sperms, downregulated genes were greatly increased, which was its main feature (Figure 4B,C).

### 2.5. Trend Analysis between DEGs of Spermatids and Sperm in Bovine and Their Homologous Genes of Transcriptome in Mouse

**Figure 5:**
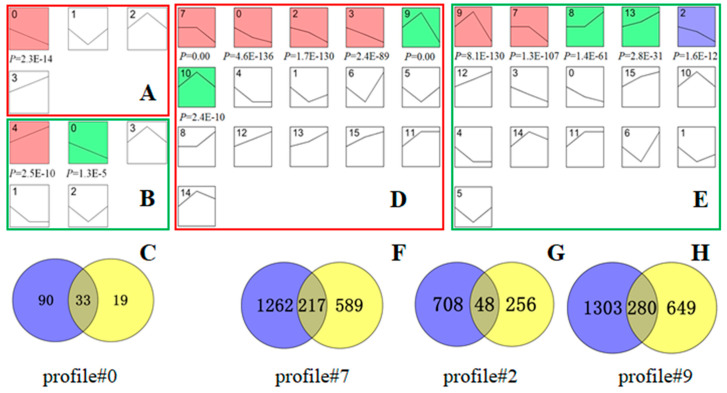
Screening of DEGs with the same trend in spermatids and sperm of bovine and mouse. The bovine (**A**,**D**) and mouse (**B**,**E**) trend charts of DEGs. Diagrams (**A**,**B**) are trend diagrams of 344 homologous genes searched in mouse sequencing data for 517 DEGs at the stage of bovine round to elongated spermatid. Diagrams (**D**,**E**) are trend diagrams of 6259 homologous genes searched in mouse sequencing data for 7279 DEGs from bovine elongated spermatid to epididymal sperm. The coloured profile graph indicates statistical significance (*p* < 0.05). After comparing (**A**,**B**), the profile#0 gene set with the same expression trend and statistical significance was screened out. (**C**) Venn diagram of the cattle and mouse profile #0 gene sets. After comparing the (**D**,**E**) graphs, the profile#7, profile#2, and profile#9 gene sets with the same expression trend and statistical significance were screened. (**F**–**H**) Venn diagrams of the profile#7, profile#2, and profile#9 gene sets of spermatids and sperm of bovine and mouse, respectively. In (**C**,**F**–**H**), the blue gene set represents the bovine homotopic gene and the yellow represents the mouse homotopic gene.

Based on the 517 DEGs from stage of round to elongated spermatids in bovine, 344 homologous genes were found in the mouse sequencing data. Figure 5A,B illustrate that profile #0 had a statistically significant number of genes assigned (*p* < 0.05), with similar trends in both species. Gene expression slightly decreased in profile #0 throughout the sperm deformation stage, and there were 123 and 52 homologous genes in bovine and mouse, respectively, and 33 overlapping genes (Figure 5C).

A total of 6259 homologous genes were found in the mouse sequencing data for the 7279 DEGs screened from the stage of elongated spermatid to epididymal sperm in bovine. Figure 5D,E show that profiles #7, #2, and #9 had statistically significant numbers of genes assigned (*p* < 0.05), and they were expressed in the same trends in the two species. Among them, the gene expression level from round to elongated spermatid was basically unchanged in profile#7, while it decreased slightly from elongated spermatid to epididymal sperm; the homologous genes for bovine and mouse were 1479 and 806, respectively, and 217 genes were overlapped. During spermiogenesis, the gene expression level in profile#2 gradually decreased, the homologous genes for bovine and mouse were 756 and 304, respectively, and 48 genes were overlapped (Figure 5G). The expression level from round to elongated spermatid was upregulated in profile#9, while the expression level from elongated spermatid to epididymal sperm was decreased, the homologous genes for bovines and mice were 1583 and 929, respectively, and 280 genes were overlapped (Figure 5H).

In summary, from the round to elongated spermatid stage, a total of 33 genes with the same homology and trend in bovine and mouse were obtained. From the elongated spermatid to the epididymal sperm, a total of 545 genes with the same homology and trend in bovine and mouse were obtained.

### 2.6. GO and KEGG Enrichment Analysis of DEGs in Bovine Homologous to Mouse with the Same Expression Trend

**Figure 6:**
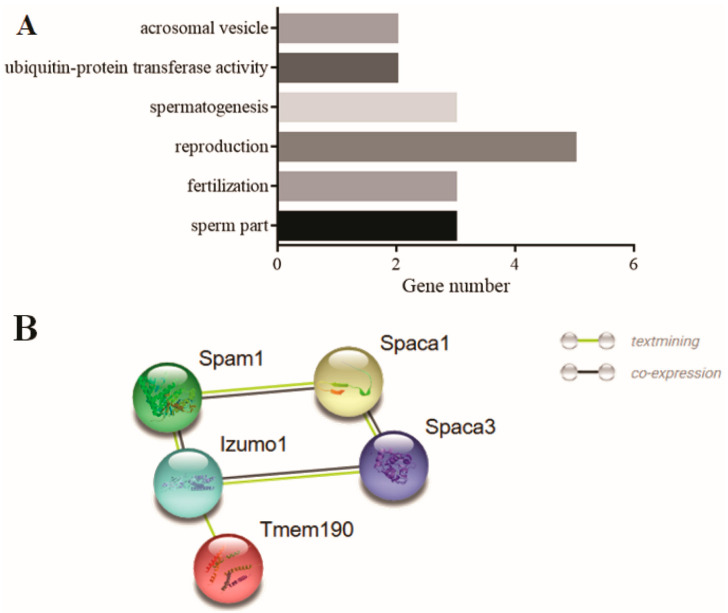

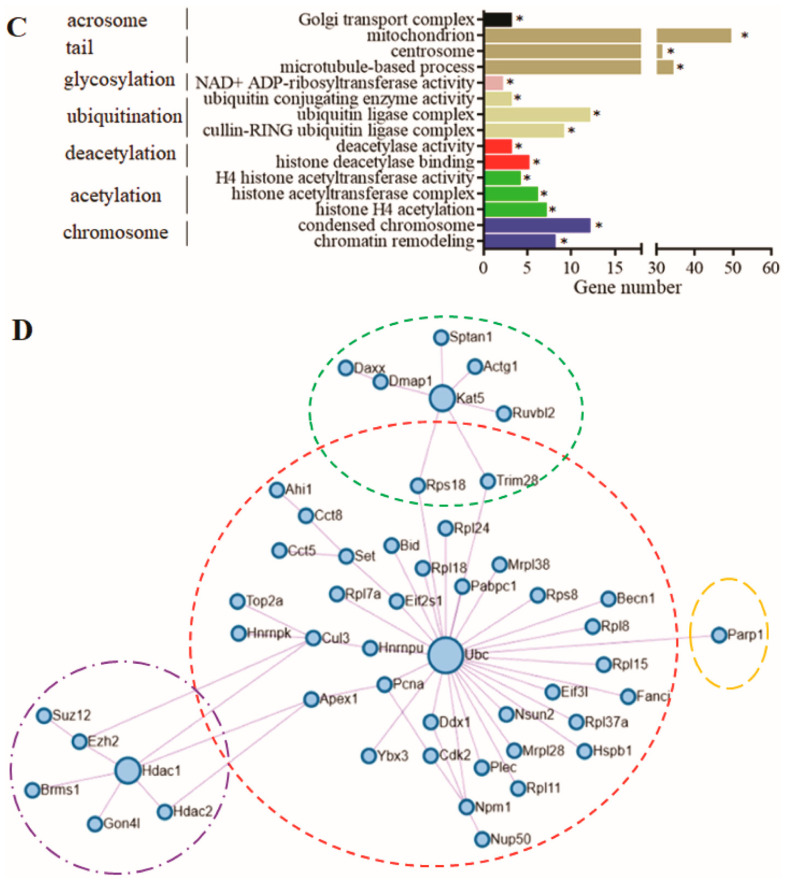
PPI and enrichment analysis results of the DEGs screened from Figure 5. (**A**) Enrichment terms in 33 DEGs from Figure 5C. (**B**) PPI regulatory network of the 33 DEGs, among which five genes constitute the core regulatory network related to sperm head formation. (**C**) Enrichment terms in 545 DEGs screened in Figure 5F–H; (**D**) the PPI regulatory network of 545 DEGs, among which, 50 genes constitute the protein epigenetic modification regulatory core network. The horizontal axes in (**A**,**C**) represent the number of genes, whereas the vertical axes represent the enriched terms. * indicates significant gene enrichment (*p* ≤ 0.05).

The enrichment analysis results displayed that (Figure 6A), among the 33 genes, the genes in the terms of acrosomal vesicle (GO: 0001669) and fertilisation (GO: 0009566) might be involved in sperm acrosome formation, the genes in terms of sperm part (GO: 0097223), reproduction (GO: 0000003), and spermatogenesis (GO: 0007283) might be involved in the deformation of the sperm head, tail, or other parts, thereby affecting sperm fertilisation ability, and the genes in terms of ubiquitin–protein transferase activity (GO: 0004842) might participate in the degradation of excess protein during sperm deformation through the protein–ubiquitinase system. PPI analysis results on DEGs selected in 3.5 from the round to elongated spermatid in bovine homologous to mouse with same trend showed that *SPAM1, SPACA1, IZUMO1, TMEM190,* and *SPACA3* constitute the core regulatory network related to the formation of sperm acrosomes (Figure 6B). 

The enrichment analysis results showed that (Figure 6C), among the 545 genes, the genes in the terms of Golgi transport complex (GO: 0017119) might be involved in sperm head formation, whereas the genes in terms of mitochondrion (GO: 0005739), centrosome (GO: 0005813), and microtubule-based processes (GO: 0007017) might be involved in sperm tail formation. The genes in terms of ubiquitin-conjugating enzyme activity (GO: 0061631), ubiquitin ligase complex (GO: 0000151), and cullin-RING ubiquitin ligase complex (GO: 0031461) might be involved in the degradation process of protein ubiquitination. The genes in terms of histone H4 acetylation (GO: 0043967), histone acetyltransferase complex (GO: 0000123), and H4 histone acetyltransferase activity (GO: 0010485) might have acetylation activity, simultaneously, and the genes in the terms of histone deacetylase binding (GO: 0042826) and deacetylase activity (GO: 0019213) might have deacetylation activity. The balance between acetylation and deacetylation could play an important role in precisely regulating and controlling various pathways in cells through protein modification. The genes in the terms of condensed chromosome (GO: 0000793) and chromatin remodelling (GO: 0006338) might be involved in sperm chromosome remodelling, resulting in a gradual decrease in the transcription level. The PPI analysis of DEGs selected in 3.5 from elongated spermatids to epididymal sperm in bovines homologous to mice with the same trend showed that 50 genes constituted the core regulatory network related to the epigenetic modification of sperm histones (Figure 6D). In this network, the genes in the red dotted circle constituted a histone ubiquitination regulatory network with ubiquitin-conjugating enzymes (UBC) as the core. The genes in the green dotted circle represent a histone acetylation regulatory network with *KAT5* as the core. The genes in the purple dotted circle represent a histone deacetylation regulatory network with *HDAC1* as the core. The *PARP1* gene in the orange dotted circle might repair DNA strand breaks caused by the replacement of histones by protamine through glycosylation.

Interestingly, PARP1, the orange dotted circle in Figure 6D, belonged to the nicotinamide adenine dinucleotide (NAD+) ADP-ribosyltransferase activity (GO: 0003950), which is a large gene family. They might repair DNA strand breaks caused by the replacement of histones by protamine via glycosylation. The DEG *ART3* in this term might have the same effect as *PARP1*.

### 2.7. ART3 Immunohistochemical Staining Results

**Figure 7:**
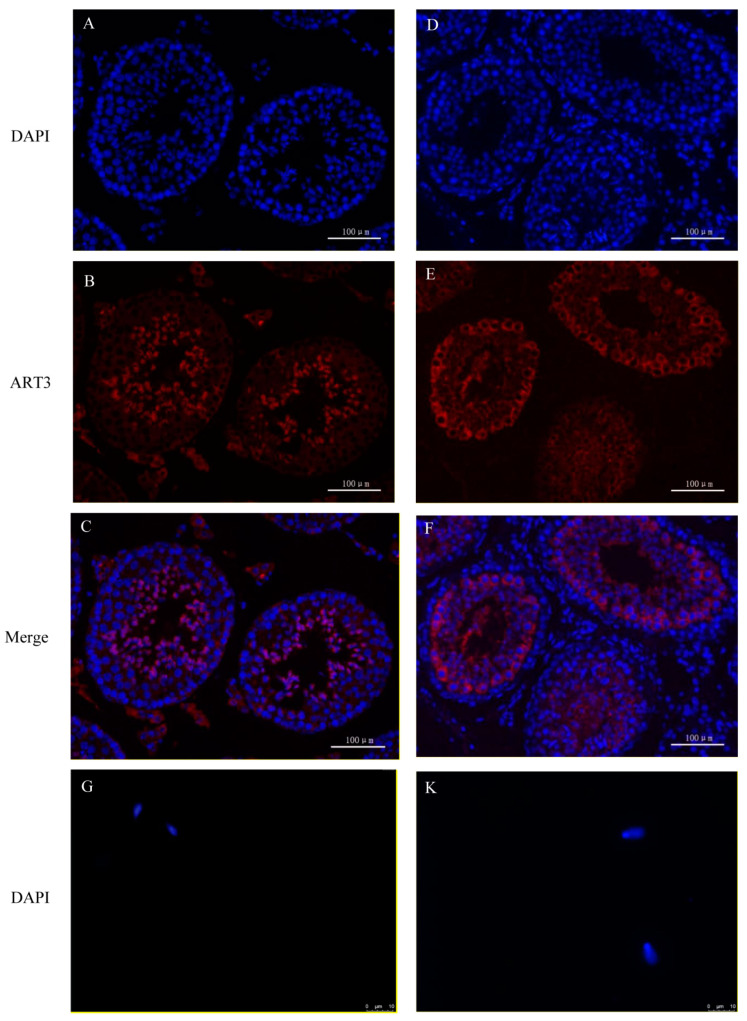

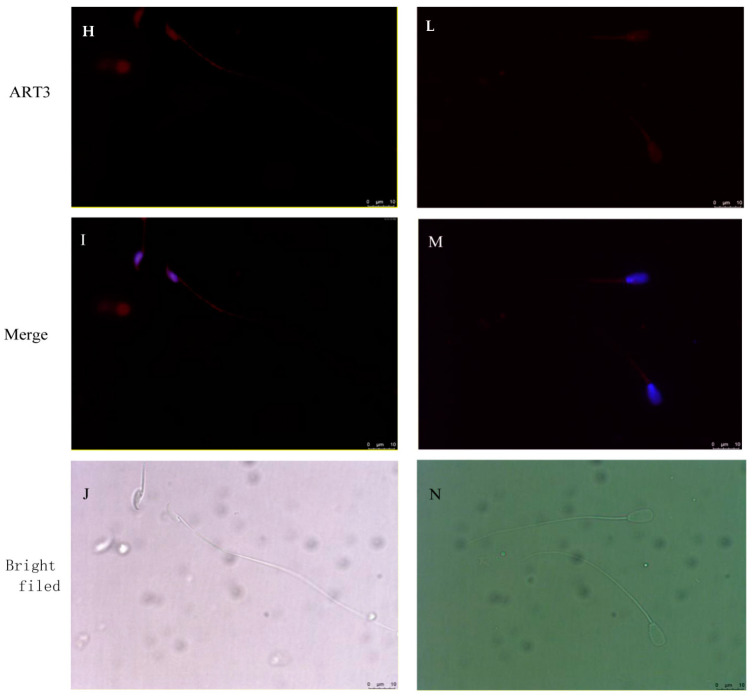

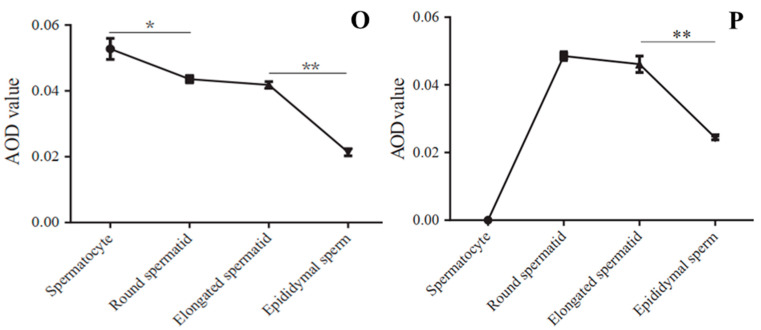
ART3 immunofluorescence staining. (**A**–**C**) Staining results of cross-sections of the seminiferous tubules of mouse testis; (**D**–**F**) staining results of cross-sections of the seminiferous tubules of bovine testis. (**G**–**J**) Staining results of mouse epididymal sperm, and (**K**–**N**) the staining results of bovine epididymal sperm. Blue represents the nucleus stained by DAPI; red represents the positive signal of the ART3 antibody. (**C**,**F**,**I**,**M**) Merged images of ART3 and nuclear staining. (**J**,**N**) The microscopic images of epididymal sperm without any staining under the bright field. (**O**,**P**) The AOD values of ART3 at different stages of spermatid development in bovine and mouse, respectively. The *y*-axis represents the AOD value, whereas the *x*-axis represents different cell types. The AOD values were represented as the mean with error bars indicating standard error of the mean (mean ± SEM). Student’s *t*-test was used to assess the statistical significance (* *p* < 0.05, ** *p* < 0.01). (**A**–**F**): ×200 magnification; scale bar = 100 μm. (**G**–**N**): ×1000 magnification; scale bar = 10 μm.

The immunofluorescence staining of ART3, a member of the ADP-ribosyltransferase family, is shown in Figure 7. First, the staining results in mouse sections are shown in Figure 7A–C. Both round and elongated spermatids in the seminiferous tubules showed red fluorescence signals of ART3 outside and blue 4’,6-Diamidino-2-phenylindole dihydrochloride (DAPI) staining signals in the nuclei. However, only the blue DAPI staining signal could be detected in other cells, such as myoid cells, spermatogonia, and spermatocytes. Further analysis revealed that the distribution of fluorescence signals on the round and elongated spermatids of mice were located towards the lumen of the seminiferous tubule; in particular, the red signal was distributed only on the outer periphery of the spermatid in the lumen. Because the head of the elongated spermatid faces the basal lamina of the seminiferous tubule, whereas the tail faces the lumen, we speculated that protein ART3 might be related to the deformation and elongation of sperm cells, formation of tails, or the shedding of cytoplasmic droplets. Further, immunofluorescence staining of mouse sperm in Figure 7G–J showed that the red fluorescent signal of ART3 appears in the tail near the head and head of sperm. In addition, the optical density value of ART3 detected in Figure 7P gradually decreased along with mouse sperm development. The average optical density (AOD) value was slightly decreased by 3.97% from round to elongated spermatids, whereas it significantly decreased (*p* < 0.05) from elongated spermatids to epididymal sperm by 47.18%.

The staining results of protein ART3 in bovine sections are shown in Figure 7D–F. The round spermatids and elongated spermatids similarly showed positive signals, consistent with results from mice. Further, immunofluorescence staining results of bovine sperm in Figure 7K–N also showed that the red fluorescent signal of ART3 appeared in the tail near the head and head of sperm. However, the periphery of spermatocytes also showed a strong and uniform ART3 signal. In addition, the AOD value of ART3 detected in Figure 7O gradually decreased, along with bovine sperm development. The AOD value significantly decreased from spermatocytes to round spermatids by 17.43% (*p* < 0.05), from elongated spermatids to epididymal sperm by 48.97% (*p* < 0.01), and from round to elongated spermatids by 4.93%.

### 2.8. Bioinformatic Analysis of Bovine ART3 Protein

The bovine ART3 protein precursor (Q3T074) contains 390 amino acids guided by the signal peptide into the Golgi apparatus and other subcellular structures after ribosomal synthesis. The first 26 signal peptide sequence is degraded by signal peptidase, and a functional mature protein is secreted by the Golgi apparatus outside the cell membrane. The molecular weight of the mature protein ART3 is 41.071 kDa, theoretical pI 5.33, which indicates a weak-acid protein. Among its 20 amino acids, leucine (Leu) accounts for the highest proportion (8.79%), whereas tryptophan (Trp) accounts for the lowest proportion (0.55%); the molecular formula of the protein is C_1841_H_2839_N_473_O_554_S_19_, its half-life is 30 min (yeast, in vivo), and its instability index is 52.05, which overall suggest an unstable protein. The Hphob/Kyte and Doolittle value of 77% of its amino acids is less than 0, indicating hydrophilicity.

According to the predicted transmembrane structure, ART3 has no transmembrane region and is completely located outside the cell membrane. Considering a signal peptide sequence in its precursor, it was speculated to be a secreted protein [13]. In addition, 56.04% of its amino acid sequence is arranged as random coil that plays a role in its secondary structure. Its *N*-terminal is mainly freely stretchable α-helices (26.65%), whereas its C-terminal is mainly a compact structure, including extended strands and β-strands (17.3%). This polar protein possesses strong *N*-terminal flexibility and relatively compact C-terminal, indicating that ART3 is likely to be an anchor protein [14].

**Figure 8:**
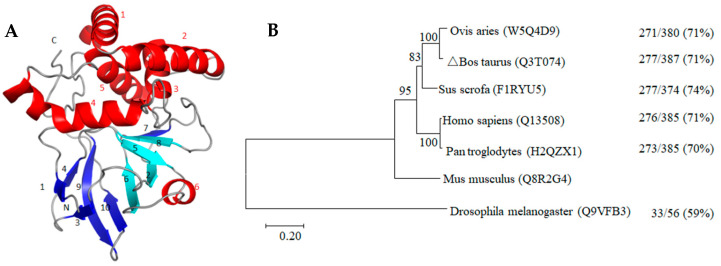
Tertiary structure (**A**) and phylogenetic tree (**B**) of bovine ART3 protein. The spirals, arrows, and loops in (**A**) represent the structures of the α-helices (Red letter number mark), β-strands (Light blue and dark blue letter number mark), and random coils, respectively. The light blue β2, β5, β6, and β8 indicate the “four-stranded β-core” conserved structure of the ART protein family. (**B**) is the phylogenetic tree constructed using the neighbour-joining method. The uniprot database number of each protein sequence is enclosed in parentheses beside the species name. The branches in the horizontal direction represent the time changes in the evolution of the pedigree. 0.20 refers to the genetic variability of the ART3 protein. The value on the right side of each branch indicates the proportion of the full-length sequence of the same or similar site, whereas that on the right indicates the proportion of the same plus similar sites of the ART3 protein of the species and the mouse to the full-length sequence.

Therefore, glycolphosphatidylinositol-anchor-site (GPI-anchor-site) prediction was performed, and omega-site position Pro was found at the C-terminus (position 359) of the ART3 protein. This site and the following hydrophobic amino acids constituted the conserved domain of the GPI-anchor protein, which further confirmed that it is indeed a GPI-anchor protein. The d1gxya template of the SCOPe2.06 database and single highest scoring were used to predict the tertiary structure of this anchored protein (Figure 8A). Based on its 215 consecutive amino acid residues with 100.0% credibility, the unique “four-stranded β-core” structure of the ART family was predicted; however, the expected arginine-specific ADP-ribosyltransferase active site motif (R-S-EXE) was not found.

In addition, the cluster analysis results of the protein sequences among species in Figure 8B shows that the *ART3* gene preceded the emergence of insects, such as *Drosophila*, with a homology of 59%. This suggests that *ART3* plays an important role in the survival of organisms. During the long biological evolution, it showed higher species conservation and was not eliminated during natural selection. Its homology among higher mammals was as high as 70–74%. Moreover, clustering bovine and sheep into one category was consistent with their closer phylogenetic relationship, suggesting that the ART3 protein may play an important role in spermiogenesis.

## 3. Discussion

### 3.1. Transcription Gradually Decreases during Spermiogenesis But Is Not Completely Suppressed

Spermiogenesis is the result of the strict regulation of related gene expression in time and space [15]. Analysis of the RNA-Seq data of bovine and mouse showed an overall decreasing gene expression during spermiogenesis. This can be attributed to the successive replacement of histones by transition proteins and protamines during spermiogenesis and the gradual condensation of the chromosomes. The haploid round spermatids are terminally differentiated cells and gradually become mature sperms, which only need to adapt to the environment to complete fertilisation and do not have any growth or development requirements [15,16]. Therefore, researchers speculated that transcription does not occur during spermiogenesis [17,18] and that internal transcripts are merely a residue from the spermatogenesis process [18,19]. However, the results of this experiment showed that even though the transcription of more genes was downregulated, there were still upregulated genes from both round spermatids to elongated spermatids and elongated spermatids to epididymal sperm. Since approximately 15% of the DNA in the mature sperm is still bound to the histone, the transcription factor may have the opportunity to combine with the specific gene sequence to initiate gene transcription [20,21]. Previous studies showed that the transcriptional expression of *Gapds* in rat sperm is significantly increased in the late stage of round spermatids [22] and that the transcriptional expression of genes, such as presidents-cup, was significantly upregulated from round spermatids into elongated spermatids in *Drosophila* [23]. Therefore, given the chromosomal constriction during spermiogenesis, transcription gradually decreases but is not completely suppressed. An in-depth study of RNA-Seq data during spermiogenesis can elucidate its physiological mechanism.

### 3.2. From Round to Elongated Spermatids, DEGs Are Mainly Related to Acrosome Formation and Fertilisation Ability

Mice are far better tools for probing the complex physiological systems shared among mammals, and the mouse genetic background has been extensively studied [24]. Homology and expression trend analyses of bovine and mouse RNA-Seq data are conducive to revealing the physiological mechanism of bovine spermiogenesis, as well as the mining and identification of important functional genes [25,26]. We also reference previous findings in human for the thoroughly study, such as genome-wide association analysis [27], location [28], and so on.

Analysis of transcriptional DEGs from round to elongated spermatids showed that DEGs are mainly involved in sperm acrosome formation and fertilisation ability, which form an interaction regulatory network with five key regulatory proteins. Among them, the membrane protein IZUMO1, located in the equatorial segment of the sperm head, may be the core regulatory gene in the network. Although Izumo^−/−^ mice can produce normal-shaped sperm that can pass through the zona pellucida, the sperm cannot fuse with the ovum [29]. The membrane protein SPACA3 located in the acrosome can interact with the oligosaccharide residue *N*-acetylglucosamine of the ovum plasma membrane to allow sperm adhesion to the ovum surface before fertilisation as preparation for crossing the ovum plasma membrane [30]. The acrosomal hyaluronidase SPAM1 enables the sperm to penetrate the hyaluronic-acid-rich cumulus cell layer surrounding the oocyte [31]. SPCA1 is located in the equatorial segment and other major parts of the sperm acrosome and plays an important role in acrosome formation and sperm–oocyte fusion. Its deletion results in abnormal acrosome morphology and male mouse sterility [32]. TMEM190, another regulatory protein of this network, is not only localised on the inner acrosomal membrane of mouse, but also co-localised with IZUMO1 in the equatorial segment of the acrosome where the sperm and oocyte fuse. However, co-immunoprecipitation experiments showed that TMEM190 and IZUMO1 have no functional interaction; however, the deletion of TMEM190 produces infertile mice [33]. Therefore, the role of TMEM190 in sperm–oocyte binding requires clarification. In summary, our findings report that the expression of genes involved in the regulatory network of acrosome formation shows a gradual downward trend during spermiogenesis and mainly reflects acrosome formation. However, these results were obtained from mice and humans; thus, further verification is warranted.

### 3.3. The Balance between Acetylation and Deacetylation Are Important to Maintain Sperm Deformation

Our DEG analysis results of the elongated spermatid to epididymal sperm showed that the replacement of histones by protamine is an important biological event. To ensure its occurrence at an appropriate time, regulatory networks of histone acetylation centred on acetyltransferase KAT5 and of histone deacetylation centred on deacetyltransferase HDAC1 were formed to jointly serve the life activity of protamine replacement of histones. The expression level of *KAT5* in mouse testes was reported to be higher than that in other organs [34]. It is located in the nuclear periphery near the round spermatid acrosome vesicle and the elongated spermatid acrosome in the seminiferous tubules of the testis. Knockout of the KAT5 gene can destroy the hyperacetylation of histone H4, and the replacement of histones by protamine is blocked, resulting in abnormal sperm [35]. Acetylation can reduce the affinity between nucleosomal histones and DNA, relaxing the chromatin structure [34]. Moreover, histone hyperacetylation ensures that transition proteins and protamine replace histones in turn in elongated spermatids, the chromosomes gradually condensing, resulting in the downregulation of transcription in sperm. One study revealed that the degree of histone hyperacetylation during spermatogenesis in infertile men is significantly lower than that in fertile men [36]. Our transcriptomic results found that the expression of *KAT5* in round and elongated spermatids was higher than in epididymal sperm, which was consistent with histone replacement by transition proteins and protamine and the downregulation of transcription during spermiogenesis. Histone acetylation lays the structural foundation for the gradual replacement of histones and guarantees gene transcription in round spermatids [37]. However, the degree of histone acetylation changes gradually in elongated spermatids, as the histones near the acrosome end are hyperacetylated whereas those near the tail are only moderately acetylated [35]. The biological significance of this change in the acetylation gradient requires further study.

Further, we found that the expression levels of deacetyltransferase HDAC1 in round and elongated spermatids were higher than in epididymal sperms. HDAC1 may act synergistically with KAT5 to ensure that protamine replaces histones at the appropriate time, which was confirmed in mouse sperm studies [38]. HDAC1 is located in the nucleus of rat spermatocytes, round spermatids, and elongated spermatids and can gradually prevent transcription by promoting histone deacetylation [39]. HDAC1 can prevent hyperacetylation of histones in the early round spermatid to ensure that histones are replaced by protamines based on physiological requirements and normal and orderly spermiogenesis [40]. Acetyltransferase activity increases in elongated spermatids, resulting in the gradual hyperacetylation of histones and its loose binding to DNA, which promotes histone replacement by protamine and leads to increased DNA fragmentation [41,42]. Inhibition of the HDAC1 expression can hinder spermatogenesis, reduce spermatogenesis, and lead to infertility [43].

Overall, the expression of HDAC1 at an appropriate time can regulate the timing and degree of the hyperacetylation of histone H4 and avoid excessive DNA fragmentation [44]. The deacetylation and acetylation regulatory networks cooperate to ensure the normal deformation of sperm.

### 3.4. Ubiquitination Plays a Critical Role in Promoting Histone Replacement by Protamine and Their Degradation

We also found that the ubiquitin regulatory network centred on UBC (ubiquitin-conjugating enzymes, E2) played an important role from elongated spermatids to epididymal sperm. UBC binds ubiquitin molecules activated by ubiquitin-activating enzyme (UBA) E1 through thioester bonds, and then interacts with ubiquitin ligase enzymes (Ub ligase) E3 to transfer the ubiquitin molecules to the excess target protein residues or organelles to degrade it during spermiogenesis [45].

Histone ubiquitination can promote the replacement of histones by protamine and the repair of DNA strand breaks, which affects spermiogenesis [42], thereby influencing sperm morphology, vitality, and quantity [46]. Therefore, some studies proposed the use of ubiquitinase to predict sperm fertilisation ability [47]. 

Ubiquitinated H2A and H2B are found in elongated spermatids, which can promote the removal and degradation of histones from the chromatin [48]. Along with the regulation of acetylation and deacetylation, the histones in sperm can be replaced and degraded in an orderly manner, leading to a gradual decrease in transcription and the formation of sperm head with dense, streamlined characteristics.

### 3.5. Members of ADP-Ribosyltransferases Can Repair DNA Strand Breaks and May Affect the Deformation of Spermatid

We also found that *PARP1* plays an important role during spermiogenesis. This gene is located in the nucleus of spermatids and can repair DNA strand breaks and maintain sperm DNA integrity. Its deletion can prevent spermatid maturation, spermatid apoptosis, and even male sterility [49]. Under the action of the TOP2B enzyme, the DNA supercoil structure changes, and the electron leakage of the mitochondria in spermatids produces excessive reactive oxygen species (ROS), which may cause DNA strand breaks [50]. DNA strand breaks activate PARP1 within a few seconds and cleave NAD+ into nicotinamide and ADP-ribose. ADP-ribose polymerises into highly negatively charged poly(ADP-ribose) (PAR) that modify histones, repair broken DNA strands, and remodel the chromatin structure [49].

Both ART3 and PARP1 are ADP-ribosyltransferases that combines with the cholesterol-rich region of the cell membrane via a GPI-anchor [51], thus exposing the “four-stranded β-core” crack region composed of β2, β5, β6, and β8. After binding this region with NAD+ [14], its active site motif transfers the ADP-ribose on NAD+ to the amino acid residues of the target protein or polymerises ADP-ribose into PAR while releasing nicotinamide.

However, using bioinformatics analysis, we found that the bovine ART3 protein has no enzymatic active site motif and thus cannot transfer ADP-ribose and cause ADP ribosylation. In addition, HEK-293-T cell transfection [28] and DC27.10 cell overexpression [51] experiments showed that ART3 does not possess ADP-ribosyltransferase activity, which confirmed our findings. ART3 was also detected in human spermatocytes and is significantly related to human nonobstructive azoospermia [27]. The transient expression of ART3 in human and bovine spermatocytes is speculated to facilitate their maturation and provide a signal source for orderly cell division [28] to promote the development of spermatocytes to haploid spermatids. However, in the later spermatocytes in humans, ART3 may fall off due to the hydrolysis of the GPI-anchored structure [28,52].

Although we detected ART3 in spermatocytes and round and elongated spermatids in bovines, its role in spermatogenesis has not yet been reported. Studies on cancer showed that ART3 can be used as a triple-negative breast cancer (TNBC) marker, and its overexpression in TNBC cells can reduce their apoptosis rate [53]. The overexpression of ART3 in melanoma cells was shown to promote cell migration [54]. The continuous division of spermatogonia is similar to the immortal proliferation of cancer cells, and the migration of spermatogenic cells is similar to the metastasis of cancer cells. Based on these similarities, we speculated that the expression patterns of genes expressed during spermiogenesis may also be similar to those in cancer cells, namely, the expression patterns of tumour–testis-associated genes, such as PRAMEL1 [55], Xrcc1 [56], and TSLC1 [57]. Immunofluorescence analysis results showed that ART3 is located around round and elongated spermatids and its polar positioning towards the direction of sperm tail formation. Based on these findings, we speculated that ART3 is involved in sperm production, polarisation, and deformation, and migration to the lumen of the seminiferous tubules and the shedding of droplets. However, experimental verification is needed to validate this.

In summary, we speculate that spermatocytes undergo meiosis and gradually differentiate into round spermatids under the induction of a signal source from ART3. Spermiogenesis begins in round spermatids, in which the formation of the acrosome is an important event, and the regulation network with *IZUMO1* as the core gene is gradually formed. Among them, SPACA3 is necessary for the adhesion reaction between sperm and oocyte. SPAM1 promotes sperm to pass through the cumulus cell layer and cooperates with IZUMO1, SPACA1, and TMEM190 to ensure sperm–oocyte fusion. In the elongated spermatid stage, spermiogenesis participates in the densification of chromosomes and the formation of head and tail. The acetylase regulatory network with *KAT5* as the core gene gradually hyperacetylates histones and loosens the structure between histones and DNA, promoting the replacement of histones by transition proteins and protamine.

Simultaneously, TOP2B changes the DNA supercoil structure, and histones H2A, H2B, and H3 are ubiquitinated. Finally, the replacement of protamine and the degradation of original histones are successfully performed. The broken DNA strands are repaired by PARP1 via glycosylation, causing the DNA strands to wrap around the smaller protamine. The deacetylation regulatory network with *HDAC1* as the core gene ensures protamine substitution and histone degradation at an appropriate time and finally leads to DNA constriction, resulting in the formation of sperm heads with dense and streamlined characteristics. ART3 promotes the polarisation, deformation, and migration of spermatids. The formation of the sperm head and the gradual decrease in gene expression are the most important events in spermiogenesis, which are achieved through the coordinated regulation of histone ubiquitination, acetylation, deacetylation, and ADP-ribosylation. In this study, we revealed important regulatory networks and their related genes, such as acrosome formation, histone ubiquitination, acetylation, and deacetylation, and critical functional genes related to sperm polarity and tail formation, such as ADP-ribosylation. Further studies are warranted to elucidate the mechanism of spermiogenesis. Our findings contribute to the improvement of the reproductive potential of bovines and the diagnosis and treatment of male infertility.

## 4. Materials and Methods

### 4.1. Ethical Statement

All animal breeding procedures and experiments were approved by the Animal Ethics Committee of the Institute of Animal Science, Chinese Academy of Agricultural Sciences (10 September 2019, CAAS; approval no, IAS2019-13).

### 4.2. RNA-Seq Analysis of Bovine Spermatids and Sperm

Our previous work conducted a transcriptomic analysis of bovine round and elongated spermatids and epididymal sperm with three biological replicates each. Holstein bovine in good health (15- to 17-months old), similar body conditions, and normal fertility were selected (*n* = 3), and their testes and epididymides were collected and placed in an ice box to be transport to the laboratory. The epididymal sperms were collected using the float-out method [15]. Briefly, 1 μL of sperm sample was diluted with PBS and counted using a haemocytometer, and the samples with an average viability of more than 50% in three visual fields were used for subsequent experiments. Meanwhile, the testis was cut into a tissue cube of approximately 1.0 × 0.6 × 0.4 cm^3^ and embedded with the optimal cutting temperature compound (OCT). It was placed in liquid nitrogen and frozen for 12–15 h, transferred to a Leica CM1950 cryostat (Leica Microsystems; Wetzlar, Germany), which had been precooled to −20 °C for 30 min, and then sliced to a thickness of 5 μm. Testis sections were filmed with a PEN2.0 membrane slide (Leica Microsystems, Wetzlar, Germany) (RNase-free) and stained with HistoGene LCM frozen section (Invitrogen, Carlsbad, CA, USA) staining kit. The Leica LMD 7000 system (Leica Microsystems, Wetzlar, Germany) was used to obtain round and elongated spermatids (Haiyang Zuo, 2016; Ren Xiaoxia, 2016; Li Xiaojun, 2018) and stored at −80 °C for future use. The samples were divided into two, one for transcriptomic sequencing and one for RT-qPCR validation.

The samples were sequenced on Illumina Hiseq 4000 platform (Illumina, San Diego, CA, USA) to generate 150-bp paired-end raw reads. Round spermatids R1, R2, and R3 had 7698, 9463, and 78.30 million raw reads, respectively, elongated spermatids E1, E2, and E3 had 7802, 7720, and 78.67 million raw reads, respectively, and the epididymal sperm M1, M2, and M3 had 7751, 7696, and 88.46 million raw reads, respectively. Raw reads from FASTQ were first processed using Perl scripts. Clean reads were obtained by removing the adaptor sequence from the reads, those containing ploy-*N*, and low-quality reads. Clean reads were aligned to the cattle reference genome (ARS-UCD1.2) using HISAT2 (v2.1.0) to build the index of the reference genome. StringTie (v2.1.2) (Johns Hopkins University, Baltimore, MD, USA) assembled the alignments into full and partial transcripts, creating multiple isoforms as necessary and estimating the expression levels of all genes and transcripts (Pertea, 2016). DEGs of the three groups were analysed as previously described (Shi et al., 2017) using the DESeq2 software (Bioconductor, open source software for bioinformatics) (http://www.bioconductor.org/). The screening threshold to satisfy the conditions for a unigene to be defined as a DEG was *p* < 0.05 and |log2 (fold change)| ≥ 1. |log2 (fold-change)| ≥ 1 means that the fold-change value must be equal or greater than 2, or the fold-change value is less than or equal to 1/2.

### 4.3. Principal Component Analysis (PCA) of Transcripts among Bovine Spermatids and Sperms

We performed PCA of the transcript data of bovine round spermatids, elongated spermatids, and epididymal sperms using the dudi.pca function in the ade4 package in R × 64 3.6.2 (Auckland University, Auckland, New Zealand).

### 4.4. Validation of RNA-Seq Data from the Bovine by RT-qPCR

Total RNA from round spermatids, elongated spermatids, and epididymal sperm samples was extracted with Trizol according to the manufacturer’s instructions. cDNA was synthesised from 1 μg total RNA using a High-Capacity cDNA Reverse Transcription Kit (Thermo Fisher Scientific, Waltham, MA, USA). Eleven genes were randomly selected for qPCR verification, and all primers (Table 2) were designed using Primer 5. β-Actin was used to normalise the qPCR expression levels [15]. The total reaction volume was 15 μL and contained 7.5 μL of 2 × SG Green qPCR Mix (SinoGene, Beijing, China), 6 μL of nuclease-free water, 0.25 μL of forward primer (10 μM), 0.25 μL of reverse primer (10 μM), and 1 μL of cDNA. qPCR was performed on a StepOnePLUS Sequence Detection System (Applied Biosystems, Inc., Carlsbad, CA, USA) using the following parameters: initial denaturation at 95 °C for 10 min; denaturation at 95 °C for 20 s; annealing at 60 °C for 30 s for 40 cycles; and dissociation at 95 °C for 15 s, 60 °C for 30 s, and 95 °C for 15 s. The 2^−△△CT^ method was used to analyse gene expression.

### 4.5. Acquisition and Analysis of Mouse RNA-Seq Data

To find functional genes with important value for life maintenance, homology analysis of bovine transcriptomic DEGs was conducted in mice with a relatively clear genetic background as a reference. RNA-seq data of mouse round spermatids, elongated spermatids, and epididymal sperms were downloaded from the GEO database. The round spermatid sample numbers were SRR3395030, SRR3395031, and SRR3395032, elongated spermatid, SRR3395024, SRR3395025, and SRR3395026, and epididymal sperm, SRR4423201, SRR4423202, and SRR4423204. An index file was constructed to create a full-length transcript. Then, transcript and gene expression were calculated.

### 4.6. Trend Analysis of Gene Expression and Screening of Genes with the Same Trend

Homologous comparison between differentially expressed genes in bovine and mouse transcriptomes was performed using the Short Time-series Expression Miner (STEM) software (v1.3.12) (Carnegie Mellon University, Pittsburgh, PA, USA) [58]. The STEM clustering method was selected, and the other parameters were set at default. STEM uses a method of analysis that takes advantage of the number of genes being large and the number of time points being few to identify statistically significant temporal expression profiles and the genes associated with these profiles [59]. Significance level was set at *p* < 0.05. After analysis of the results, we acquired a statistically significant profile that had the same expression trend in both bovine and mice. Then, the common genes between the two species were screened.

### 4.7. GO Term and KEGG Pathway Enrichment Analysis

GO terms and KEGG enrichment analysis of the selected common genes were performed using the Webgestalt2017 software (University of Tennessee-Oak Ridge National Laboratory, Oak Ridge, TN, USA) (http://www.webgestalt.org/). For the enrichment analysis, the software was set to the over-representation analysis (ORA), and other parameters were as default. GO terms and KEGG pathways with corrected *p*-values less than 0.05 were considered significantly enriched.

### 4.8. Protein–Protein Interaction Network (PPI network) Analysis

Based on the BioGrid database [60], Metascape online software (Genomics Institute of the Novartis Research Foundation, Infectious and Inflammatory Disease Center, and University of California, Berkeley, CA, USA) (http://metascape.org) was used to perform PPI analysis on the selected common genes to identify all the differentially expressed genes with related regulatory relationships. Then, we used the molecular complex detection (MCODE) algorithm to screen modules with important biological functions in PPI [61].

### 4.9. Immunofluorescence Staining of Bovine and Mouse Sperm and Testes

Bovine and mouse testis tissue cubes were soaked in 4% paraformaldehyde, fixed for 48 h, and successively dehydrated in a series of alcohol solutions (70%, 85%, 95%, 100%, and 100% alcohol solutions) for 2 h. Then, the tissue cubes were successively immersed in a mixture of xylene and alcohol (1:1), xylene, and xylene for 1 h, embedded in paraffin, and cut into 4 μm thick sections for immunofluorescence analysis. Following deparaffinisation and rehydration, the sections were subjected to heat-induced antigen retrieval by heating to 100 °C in sodium citrate buffer (10 mM sodium citrate, 0.05% Tween 20, pH 6.0) for 10 min. After washing in PBS, the sections were blocked in 5% bovine serum albumin (BSA) for 45 min at room temperature (RT) and incubated with anti-ART3 (1:100, sc-515157, Santa Cruz, Dallas, TX, USA) overnight at 4 °C. Following the washing step, sections were further incubated in the dark with CoraLite594-conjugated goat anti-mouse IgG (H + L) (1:500, SA00013-3, Proteintech, Rosemont, IL, USA) for 2 h at RT. After secondary antibody incubation, sections were washed in phosphate buffer solution (PBS), and nuclei were counterstained with DAPI (KGA215-50, KeyGEN) for 8 min at RT. Sections were washed in PBS, and the coverslips were mounted. Images were captured using an Olympus BX 45 microscope (Olympus, Tokyo, Japan) with objective parameters of the UplanSApo 20 × 0.75 objective and 200× images are shown.

The epididymal sperm were collected using the float-out method [15]. Sperm samples of 1 μL were diluted with PBS and counted with a haemocytometer, and the sperm samples with an average viability of more than 50% in 3 visual fields were used for subsequent experiments. The diluted sperm solution was placed in 15-mL centrifuge tube and centrifuged at 300 rpm for 5 min, and the supernatant was carefully aspirated with a 200 μL pipette and discarded. Then, 10 mL of 4% paraformaldehyde solution was added to the centrifuge tube, fixed for 3 h, and centrifuged at 300 rpm for 5 min. After removing the supernatant, 0.1 M sucrose solution was added at room temperature for 3 h, and a pipette was used to smear it on the slices predropped with 1% paraformaldehyde and 0.15% TritonX-100. After air drying, the slices were washed three times in PBS for 5 min each to remove excess paraformaldehyde, sucrose, and TritonX-100. The slices were permeated in ice-cold methanol (−20 °C) for 5 min, and washed in PBS, and antigen retrieval was achieved by boiling in sodium citrate buffer for 20 min. After washing in PBS, the sections were blocked in 5% bovine serum albumin (BSA) for 45 min at room temperature (RT) and incubated with anti-ART3 (1:100, sc-515157, Santa Cruz, Dallas, TX, USA) overnight at 4 °C. Following the washing step, sections were further incubated in the dark with CoraLite594-conjugated goat anti-mouse IgG (H + L) (1:500, SA00013-3, Proteintech, Rosemont, IL, USA) for 2 h at RT. After the secondary antibody incubation, sections were washed in PBS, and nuclei were counterstained with DAPI (KGA215-50, KeyGEN) for 8 min at RT. Sections were washed in PBS, and coverslips were mounted. Images were captured using an Olympus BX 45 microscope with objective parameters of the UplanSApo 100 × 1.35 objective. Representative 1000× magnified images are shown.

### 4.10. Measurement of Optical Density

The Pannoramic section scanner was used to image the tissue section. CaseViewer2.2 scanning software (The digital pathology company, Hungary) was used to select the target area of the tissue for imaging. During imaging, the whole field of vision was filled as much as possible to ensure consistent background light for each photo. After imaging, Image-Pro Plus 6.0 analysis software (CAD/CAM Services Inc., Celina, TX, USA) was used to measure the positive integrated optical density (IOD) values of the three fields in each tissue slice expressed as pixel area. The corresponding positive pixel area (area) was calculated as follows: AOD value = IOD/Area. GraphPad Prism6 software (GraphPad, San Diego, CA, USA) was used to generate a line graph analysis of the AOD value.

### 4.11. Bioinformatics Analysis of DEG ART3

The bovine ART3 protein precursor sequence was downloaded from the UniProt database (https://www.uniprot.org/), while the online server SignalP-5.0 Server was used to predict the protein signal peptide sequence and cut it off to obtain the mature ART3 protein sequence. The online server ExPASy-ProtParam (https://web.expasy.org/protparam/) was used to predict the physical and chemical properties of the mature ART3 protein sequence. The online server ExPasy-ProtScale (https://web.expasy.org/protscale/) was used to predict its hydrophilicity and hydrophobicity. At the same time, the ART3 protein structure was predicted. First, the online server transmembrane hidden Markov model 2.0 (TMHMM 2.0) Server (http://www.cbs.dtu.dk/services/TMHMM/) was used to predict its transmembrane structure, then using online servers self-optimized prediction method with alignment (SOPMA) (https://npsa-prabi.ibcp.fr/cgi-bin/npsa_automat.pl?page=npsa_sopma.html) and Phyre2 (http://www.sbg.bio.ic.ac.uk/phyre2/html/page.cgi?id=index) predicted its secondary structure and tertiary structure, and the online server PredGPI (http://gpcr.biocomp.unibo.it/predgpi/pred.htm) was used to predict its GPI-anchor structure. Finally, Molecular Evolutionary Genetics Analysis 7 (MEGA 7) (Temple University, Philadelphia, PA, USA; Tokyo Metropolitan University, Tokyo, Japan) (https://www.megasoftware.net/) software was used to construct a phylogenetic tree based on the ART3 protein sequence.

### 4.12. Statistical Analysis

All experiments were performed at least three times. Data were analysed using one-way ANOVA or unpaired two-tailed *t*-test. *p*-values < 0.05 were considered to indicate statistically significant differences. All data were represented using the mean ± standard deviation (mean ± SD). Analyses were performed using Microsoft Excel 2010 (Microsoft, Redmond, WA, USA) and GraphPad Prism 6.0 and R_x64 3.6.2 (Auckland University, Auckland, New Zealand).

## Figures and Tables

**Table 1:** Sequencing output of bovine spermatid and sperm samples.

Samples	R1	R2	R3	E1	E2	E3	M1	M2	M3
Raw reads ^1^ (million)	76.983	94.627	78.303	78.018	77.196	78.670	77.515	76.957	88.457
Clean reads ^2^ (million)	72.947	72.556	71.185	73.697	71.376	71.570	66.614	71.147	79.564
Clean reads rate ^3^ (%)	94.8	76.7	90.9	94.5	92.5	91.0	85.9	92.5	90.0
Clean Q30 bases rate ^4^ (%)	94.0	93.1	93.2	94.3	94.0	93.8	91.0	92.6	92.6
Mapped reads ^5^ (million)	64.529	63.465	61.395	65.593	63.068	61.930	59.316	63.354	71.666
Mapping rate ^6^ (%)	88.4	87.5	86.2	89.0	88.4	86.5	89.0	89.0	90.0

Note: ^1^ Raw reads: Total number of sequences from original sequencing; ^2^ Clean reads: Number of high-quality sequence reads after filtration; ^3^ Clean reads rate: Ratio of the high-quality read number to the original read number; ^4^ Clean Q30 base rate (%): Error probability of base calling during sequencing. After filtration, the sequencing quality value of clean reads is more than 30, that is, the proportion of bases with an error rate is less than 0.1%; ^5^ Mapped reads: Number of clean reads sequences aligned to the reference genome; ^6^ Mapping rate: Ratio of clean reads sequence number to reference genome. R: round spermatid; E: elongated spermatid; M: epididymal sperm.

**Table 2:** Primers used for qPCR analysis of bovine samples.

Gene Name	Primer Sequence (5′-3′)	Size (bp)
*ACTIN*	Forward Primer: ATCCTGCGGCATTCACGAA	150
	Reverse Primer: TGCCAGGGCAGTGATCTCTT	
*MIGA2*	Forward Primer: CACGTTAGCCCTGTCCTAGC	120
	Reverse Primer: TCGAACATGTCGGTCAGGTA	
*CREM*	Forward Primer: AGGAAGAGGGAACACCACCT	104
	Reverse Primer: GATTGTTCCACCTTGGGCTA	
*YBX2*	Forward Primer: AGGAATGACACCAAGGAGGA	111
	Reverse Primer: GACATCGAACTCCACGGTCT	
*SDHA*	Forward Primer: TAAACCAAATGCTGGGGAAG	109
	Reverse Primer: CTGCATCGACTTCTGCATGT	
*ART3*	Forward Primer: CCGGATGAAAAACCTGAAGA	116
	Reverse Primer: AGGAGCATTTTGCCAGAAGA	
*DNAL1*	Forward Primer: TCAAAGAAGCCCTAGCCAGA	125
	Reverse Primer: AGAAAGCGTGGACAAGGATG	
*PRKAR1A*	Forward Primer: CGGTTTCCTTTATTGCTGGA	110
	Reverse Primer: TGCCCATTCATTGTTGACAT	
*TEKT2*	Forward Primer: ACGAGACCAACAACCAGACC	112
	Reverse Primer: TCCGTCAGACACTTGTCCAG	
*OAZ3*	Forward Primer: AGAAGATGCTGCCTTGCTGT	98
	Reverse Primer: AGGGACTCAGGAGCACTGAA	
*HIP1*	Forward Primer: GATCGAGGACACAGACAGCA	128
	Reverse Primer: CCAGTTTCTGACGCTCCTTC	
*LMTK2*	Forward Primer: CTGCTGGCTGTCACCAGATA	107
	Reverse Primer: GCTGCTCAAAGTCCACTTCC	

## Data Availability

The data presented in this study are available on request from the corresponding author. Data sharing is not applicable to this article.

## Abbreviations

DEGs	Differentially expressed genes
GO	Gene Ontology
KEGG	Kyoto Encyclopaedia of Genes and Genomes
RT	Room temperature
RT-qPCR	Quantitative real-time RT PCR
ORA	Over-representation analysis
MCODE	Molecular complex detection
PPI	Protein–protein interaction
ROS	Reactive oxygen species
PCA	Principal component analysis
IOD	Integrated optical density
AOD	Average optical density

## References

[1] Schenk J.L. (2018). Review: Principles of maximizing bull semen production at genetic centers. Animal.

[2] Fair S., Lonergan P. (2018). Review: Understanding the causes of variation in reproductive wastage among bulls. Animal.

[3] Schlegel P.N. (2009). Evaluation of male infertility. Minerva. Ginecol..

[4] Simon L., Emery B.R., Carrell D.T. (2017). Review: Diagnosis and impact of sperm DNA alterations in assisted reproduction. Best Pract. Res. Obstet. Gynaecol..

[5] Hamze J.G., Sanchez J.M., O’Callaghan E., McDonald M., Bermejo-Alvarez P., Romar R., Lonergan P., Jimenez-Movilla M. (2020). JUNO protein coated beads: A potential tool to predict bovine sperm fertilizing ability. Theriogenology.

[6] Devlin D.J., Zaneveld S.A., Nozawa K., Han X., Moye A.R., Liang Q., Harnish J.M., Matzuk M.M., Chen R. (2020). Knockout of mouse receptor accessory protein 6 leads to sperm function and morphology defects. Biol. Reprod..

[7] Evenson D.P., Wixon R. (2008). Data analysis of two in vivo fertility studies using Sperm Chromatin Structure Assay-derived DNA fragmentation index vs. pregnancy outcome. Fertil. Steril..

[8] Manterola M., Brown T.M., Oh M.Y., Garyn C., Gonzalez B.J., Wolgemuth D.J. (2018). BRDT is an essential epigenetic regulator for proper chromatin organization, silencing of sex chromosomes and crossover formation in male meiosis. PLoS Genet..

[9] Shang E., Nickerson H.D., Wen D., Wang X., Wolgemuth D.J. (2007). The first bromodomain of Brdt, a testis-specific member of the BET sub-family of double-bromodomain-containing proteins, is essential for male germ cell differentiation. Development.

[10] Lu L.Y., Wu J., Ye L., Gavrilina G.B., Saunders T.L., Yu X. (2010). RNF8-dependent histone modifications regulate nucleosome removal during spermatogenesis. Dev. Cell.

[11] Cheon Y.-P., Kim C.-H. (2015). Impact of glycosylation on the unimpaired functions of the sperm. Clin. Exp. Reprod. Med..

[12] Chung J.-J., Miki K., Kim D., Shim S.-H., Shi H.F., Hwang J.Y., Xinjiang Cai Y.I., Zhuang X., Clapham D.E. (2017). CatSperζ regulates the structural continuity of sperm Ca2+ signaling domains and is required for normal fertility. eLife.

[13] Petitot A.S., Dereeper A., Agbessi M., Silva C.D., Guy J., Ardisson M., Fernandez D. (2016). Dual RNA-seq reveals Meloidogyne graminicola transcriptome and candidate effectors during the interaction with rice plants. Mol. Plant. Pathol..

[14] Mueller-Dieckmann C., Ritter H., Haag F., Koch-Nolte F., Schulz G.E. (2002). Structure of the Ecto-ADP-ribosyl Transferase ART2.2 from Rat. J. Mol. Biol..

[15] Zuo H., Zhang J., Zhang L., Ren X., Wang D. (2016). Transcriptomic Variation during Spermiogenesis in Mouse Germ Cells. PLoS ONE.

[16] Wang Q., Lu W., Yang J., Jiang L., Zhang Q., Kan X., Yang X. (2018). Comparative transcriptomics in three Passerida species provides insights into the evolution of avian mitochondrial complex I. Comp. Biochem. Physiol. Part D Genom. Proteom..

[17] D’Occhio M.J., Hengstberger K.J., Johnston S.D. (2007). Biology of sperm chromatin structure and relationship to male fertility and embryonic survival. Anim. Reprod. Sci..

[18] Ren X., Chen X., Wang Z., Wang D. (2017). Is transcription in sperm stationary or dynamic?. J. Reprod. Dev..

[19] Fischer B.E., Wasbrough E., Meadows L.A., Randlet O., Dorus S., Karr T.L., Russell S. (2012). Conserved properties of Drosophila and human spermatozoal mRNA repertoires. Proc. Biol. Sci..

[20] Kramer J.A., Mccarrey J.R., Djakiew D., Krawetz S.A. (2000). Human spermatogenesis as a model to examine gene potentiation. Mol. Reprod. Dev..

[21] Wykes S.M., Krawetz S.A. (2003). The Structural Organization of Sperm Chromatin. J. Biol. Chem..

[22] Welch J.E., Barbee R.R., Magyar P.L., Bunch D.O., O’Brien D.A. (2006). Expression of the spermatogenic cell-specific glyceraldehyde 3-phosphate dehydrogenase (GAPDS) in rat testis. Mol. Reprod. Dev..

[23] Barreau C., Benson E., Gudmannsdottir E., Newton F., White-Cooper H. (2008). Post-meiotic transcription in Drosophila testes. Development.

[24] Breschi A., Gingeras T.R., Guigó R. (2017). Comparative transcriptomics in human and mouse. Nat. Rev. Genet..

[25] Ghandhi S.A., Sinha A., Markatou M., Amundson S.A. (2011). Time-series clustering of gene expression in irradiated and bystander fibroblasts: An application of FBPA clustering. BMC Genom..

[26] Zheng Y., Li X., Huang Y., Jia L., Li W. (2018). Time series clustering of mRNA and lncRNA expression during osteogenic differentiation of periodontal ligament stem cells. Peerj.

[27] Lin F., Jiang L., Yang H., Yang X., Wu J., Huang X., Ni W. (2015). Association of polymorphisms in ART3 gene with male infertility in the Chinese population. Int. J. Clin. Exp. Med..

[28] Friedrich M., Grahnert A., Paasch U., Tannapfel A., Koch-Nolte F., Hauschildt S. (2006). Expression of toxin-related human mono-ADP-ribosyltransferase 3 in human testes. Asian J. Androl..

[29] Inoue N., Ikawa M., Isotani A., Okabe M. (2005). The immunoglobulin superfamily protein Izumo is required for sperm to fuse with eggs. Nature.

[30] Ito C., Toshimori K. (2016). Acrosome markers of human sperm. Anat. Sci. Int..

[31] Saindon A., Leclerc P. (2018). SPAM1 and PH-20 are two gene products expressed in bovine testis and present in sperm. Reproduction.

[32] Kishida K., Harayama H., Kimura F., Murakami T. (2016). Individual differences in the distribution of sperm acrosome-associated 1 proteins among male patients of infertile couples; their possible impact on outcomes of conventional in vitro fertilization. Zygote.

[33] Nishimura H., Gupta S., Myles D.G., Primakoff P. (2011). Characterization of mouse sperm TMEM190, a small transmembrane protein with the trefoil domain: Evidence for co-localization with IZUMO1 and complex formation with other sperm proteins. Reproduction.

[34] Thomas T., Loveland K.L., Voss A.K. (2007). The genes coding for the MYST family histone acetyltransferases, Tip60 and Mof, are expressed at high levels during sperm development. Gene Exp. Patterns.

[35] Dong Y., Isono K.I., Ohbo K., Endo T.A., Ohara O., Maekawa M., Toyama Y., Ito C., Toshimori K., Helin K. (2017). EPC1/TIP60-mediated histone acetylation facilitates spermiogenesis in mice. Mol. Cell Biol..

[36] Sonnack V., Failing K., Bergmann M., Steger K. (2002). Expression of hyperacetylated histone H4 during normal and impaired human spermatogenesis. Andrologia.

[37] Steger K. (2001). Haploid spermatids exhibit translationally repressed mRNAs. Anat. Embryol..

[38] Kim J., Kim J.-H., Jee B.-C., Suh C.-S., Kim S.-H. (2015). Is There a Link Between Expression Levels of Histone Deacetylase/Acetyltransferase in Mouse Sperm and Subsequent Blastocyst Development?. Reprod. Sci..

[39] Almabhouh F.A., Singh H.J. (2018). Adverse effects of leptin on histone-to-protamine transition during spermatogenesis are prevented by melatonin in Sprague-Dawley rats. Andrologia.

[40] Hazzouri M., Pivot-Pajot C., Faure A.K., Usson Y., Pelletier R., Sele B., Khochbin S., Rousseaux S. (2000). Regulated hyperacetylation of core histones during mouse spermatogenesis: Involvement of histone deacetylases. Eur. J. Cell Biol..

[41] Min Y., Mi-Jeong K., Sena L., Eunyoung C., Ki-Young L. (2018). Inhibition of TRAF6 ubiquitin-ligase activity by PRDX1 leads to inhibition of NFKB activation and autophagy activation. Autophagy.

[42] Kim R.N., Kim D.-W., Choi S.-H., Chae S.-H., Nam S.-H., Kim D.-W., Aeri Kim A.K., Park K.-H., Lee Y.S., Hirai M. (2011). Major chimpanzee-specific structural changes in sperm development-associated genes. Funct. Integr. Genom..

[43] González B., Pantoja C.R.G., Sosa M.H., Vitullo A.D., Bisagno V., González C.R. (2018). Cocaine alters the mouse testicular epigenome with direct impact on histone acetylation and DNA methylation marks. Reprod. Biomed. Online.

[44] Gill-Sharma M.K., Choudhuri J., Ansari M.A., D’Souza S. (2012). Putative molecular mechanism underlying sperm chromatin remodelling is regulated by reproductive hormones. Clin. Epigenetics.

[45] Hanpude P., Bhattacharya S., Dey A.K., Maiti T.K. (2015). Deubiquitinating enzymes in cellular signaling and disease regulation. IUBMB Life.

[46] Sutovsky P. (2018). Review: Sperm–oocyte interactions and their implications for bull fertility, with emphasis on the ubiquitin–proteasome system. Animal.

[47] Muratori M., Marchiani S., Criscuoli L., Fuzzi B., Tamburino L., Dabizzi S., Pucci C., Evangelisti P., Forti G., Noci I. (2007). Biological meaning of ubiquitination and DNA fragmentation in human spermatozoa. Soc. Reprod. Fertil. Suppl..

[48] Bao J., Bedford M.T. (2016). Epigenetic regulation of the histone-to-protamine transition during Spermiogenesis. Reproduction.

[49] Agarwal A., Mahfouz R.Z., Sharma R.K., Sarkar O., Mangrola D., Mathur P.P. (2009). Potential biological role of poly (ADP-ribose) polymerase (PARP) in male gametes. Reprod. Biol. Endocrinol..

[50] Meyer-Ficca M.L., Lonchar J.D., Ihara M., Meistrich M.L., Austin C.A., Meyer R.G. (2011). Poly(ADP-Ribose) Polymerases PARP1 and PARP2 Modulate Topoisomerase II Beta (TOP2B) Function during Chromatin Condensation in Mouse Spermiogenesis. Biol. Reprod..

[51] Leutert M., Menzel S., Braren R., Rissiek B., Hopp A.K., Nowak K., Bisceglie L., Gehrig P., Li H., Zolkiewska A. (2018). Proteomic Characterization of the Heart and Skeletal Muscle Reveals Widespread Arginine ADP-Ribosylation by the ARTC1 Ectoenzyme. Cell Rep..

[52] Menzel S., Rissiek B., Bannas P., Jakoby T., Miksiewicz M., Schwarz N., Nissen M., Haag F., Tholey A., Koch-Nolte F. (2015). Nucleotide-Induced Membrane-Proximal Proteolysis Controls the Substrate Specificity of T Cell Ecto-ADP-Ribosyltransferase ARTC2.2. J. Immunol..

[53] Tan L., Song X., Sun X., Wang N., Sun Z. (2016). ART3 regulates triple-negative breast cancer cell function via activation of Akt and ERK pathways. Oncotarget.

[54] He J., Li Y., Wang Y., Zhang H., Ge S., Fan X. (2018). Targeted silencing of the ADP-ribosyltransferase 3 gene inhibits the migration ability of melanoma cells. Oncol. Lett..

[55] Mistry B.V., Zhao Y., Chang T.C., Hiroshi Y., Mitsuru C., Jon O., Francisco D., Liu W.S., John C.A. (2013). Differential Expression of PRAMEL1, a Cancer/Testis Antigen, during Spermatogenesis in the Mouse. PLoS ONE.

[56] Tebbs R.S., Thompson L.H., Cleaver J.E. (2003). Rescue of Xrcc1 knockout mouse embryo lethality by transgene-complementation. DNA Repair..

[57] Van der Weyden L., Arends M.J., Chausiaux O.E., Ellis P.J., Lange U.C., Surani M.A., Affara N., Murakami Y., Adams D.J., Bradley A. (2006). Loss of TSLC1 causes male infertility due to a defect at the spermatid stage of spermatogenesis. Mol. Cell Biol..

[58] Ernst J., Bar-Joseph Z. (2006). STEM: A tool for the analysis of short time series gene expression data. BMC Bioinform..

[59] Ernst J., Nau G.J., Bar-Joseph Z. (2005). Clustering short time series gene expression data. Bioinformatics.

[60] Stark C., Breitkreutz B.J., Reguly T., Boucher L., Breitkreutz A., Tyers M. (2006). BioGRID: A general repository for interaction datasets. Nucleic Acids Res..

[61] Bandettini W.P., Kellman P., Mancini C., Booker O.J., Vasu S., Leung S.W., Wilson J.R., Shanbhag S.M., Chen M.Y., Arai A.E. (2012). MultiContrast Delayed Enhancement (MCODE) improves detection of subendocardial myocardial infarction by late gadolinium enhancement cardiovascular magnetic resonance: A clinical validation study. J. Cardiovasc. Magn. Reson..

